# Supplementary material for: Analysis of SARS-CoV-2 isolates, namely the Wuhan strain, Delta variant, and Omicron variant, identifies differential immune profiles
Source: Microbiol Spectr. 2023 Sep 7;11(5):e01256-23. doi: 10.1128/spectrum.01256-23 (PMC10581158; doi:10.1128/spectrum.01256-23)
Supplement: Supplemental Figures — 1-3 [file spectrum.01256-23-s0001.pdf]

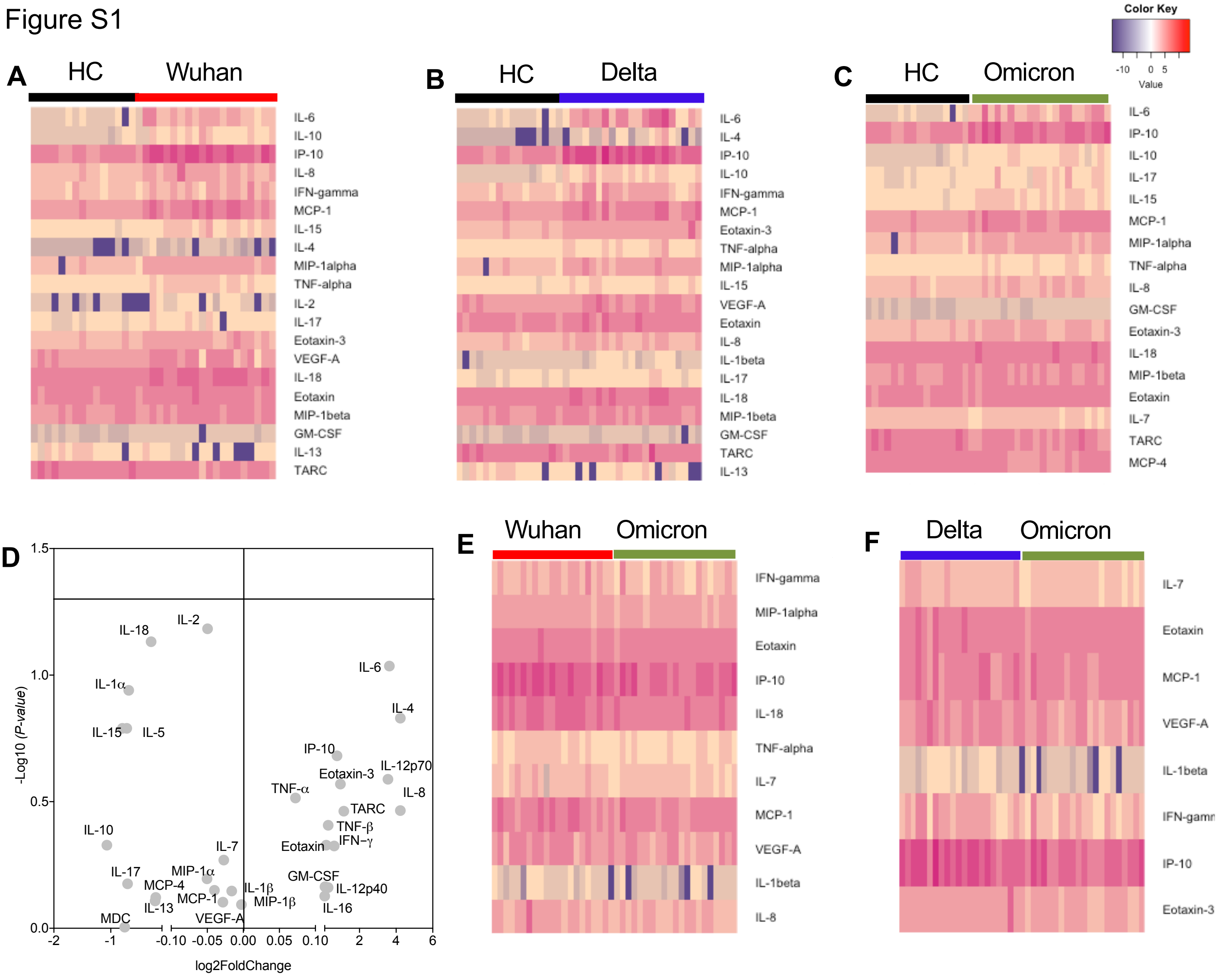

Figure S1. A Heaptmaps showing the concentration of indicated cytokines/chemokines per individual in HCs vs infected individuals with the Wuhan strain. **B** Heaptmaps showing the concentration of indicated cytokines/chemokines per individual in HCs vs infected individuals with the Delta variant. **C** Heaptmaps showing the concentration of indicated cytokines/chemokines per individual in HCs vs infected individuals with the Omicron variant. **D** The volcano plot illustrating the magnitude and difference in cytokine/chemokine concentrations in individuals infected with the Wuhan strain vs the Delta variant. **E** Heaptmaps showing the concentration of indicated cytokines/chemokines per individual in infected individuals with the Wuhan strain vs the Omicron variant. **F** Heaptmaps showing the concentration of indicated cytokines/chemokines per individual in infected individuals with the Delta vs Omicron variant.

Figure S2

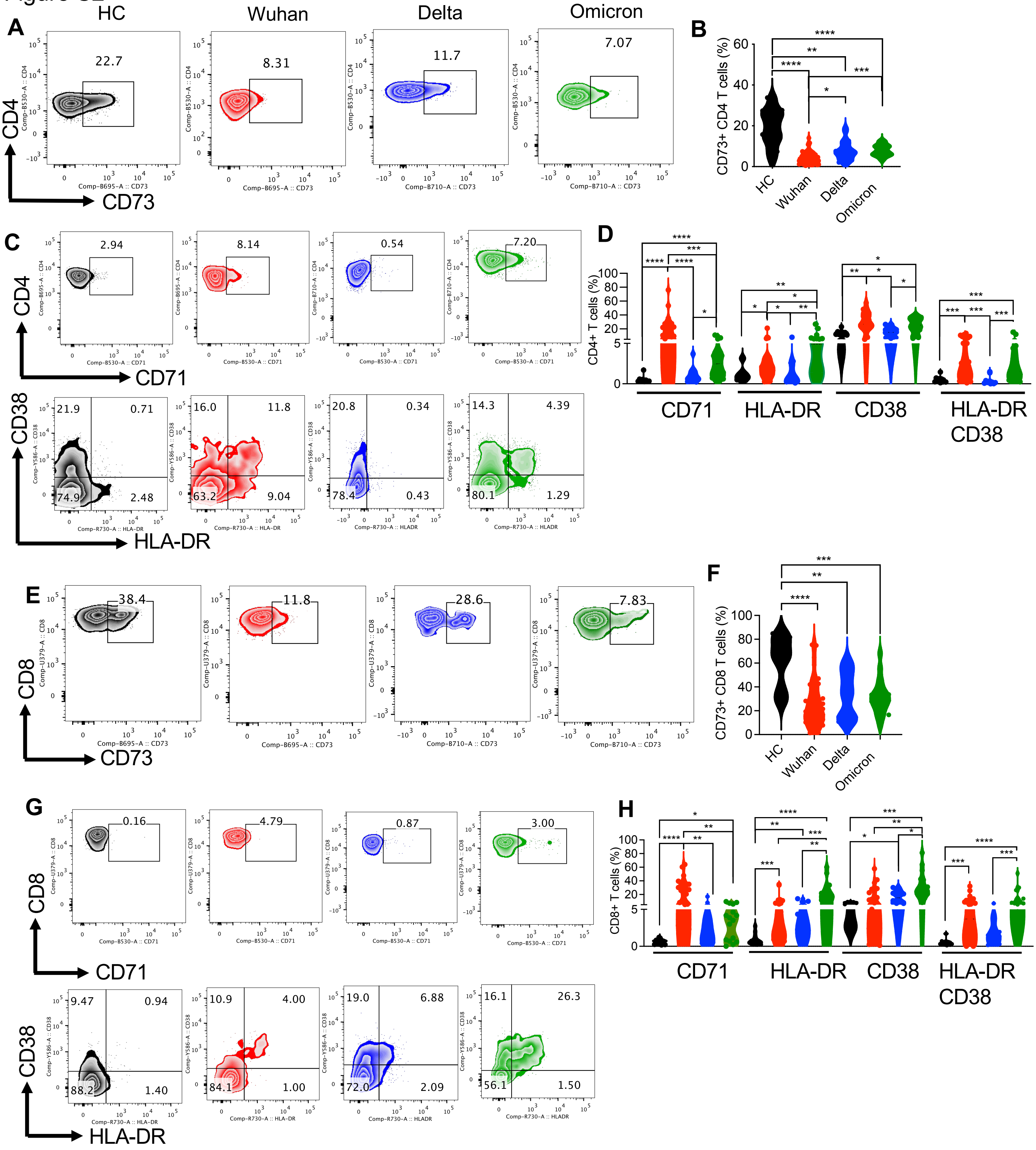

Figure S2. **A** Representative flow cytometry plots, and **B** cumulative data of the percentages of CD73 expressing $CD4^+$ T cells in HCs vs individuals infected with the Wuhan strain, Delta, and Omicron variants. **C** Representative flow cytometry plots, and **D** cumulative data showing percentages of $CD4^+CD71^+$, $CD4^+HLA\text{-}DR^+$, $CD4^+CD38^+$, and $CD4^+CD38^+HLA\text{-}DR^+$ T cells in HCs vs individuals infected with the Wuhan strain and the Delta/Omicron variants. **E** Representative flow cytometry plots, and **F** cumulative data of the percentages of CD73 expressing $CD8^+$ T cells in HCs vs individuals infected with the Wuhan strain, Delta, and Omicron variants. **G** Representative flow cytometry plots, and **H** cumulative data showing percentages of $CD8^+CD71^+$, $CD8^+HLA\text{-}DR^+$, $CD8^+CD38^+$, and $CD8^+CD38^+HLA\text{-}DR^+$ T cells in HCs vs individuals infected with the Wuhan strain and the Delta/Omicron variants.

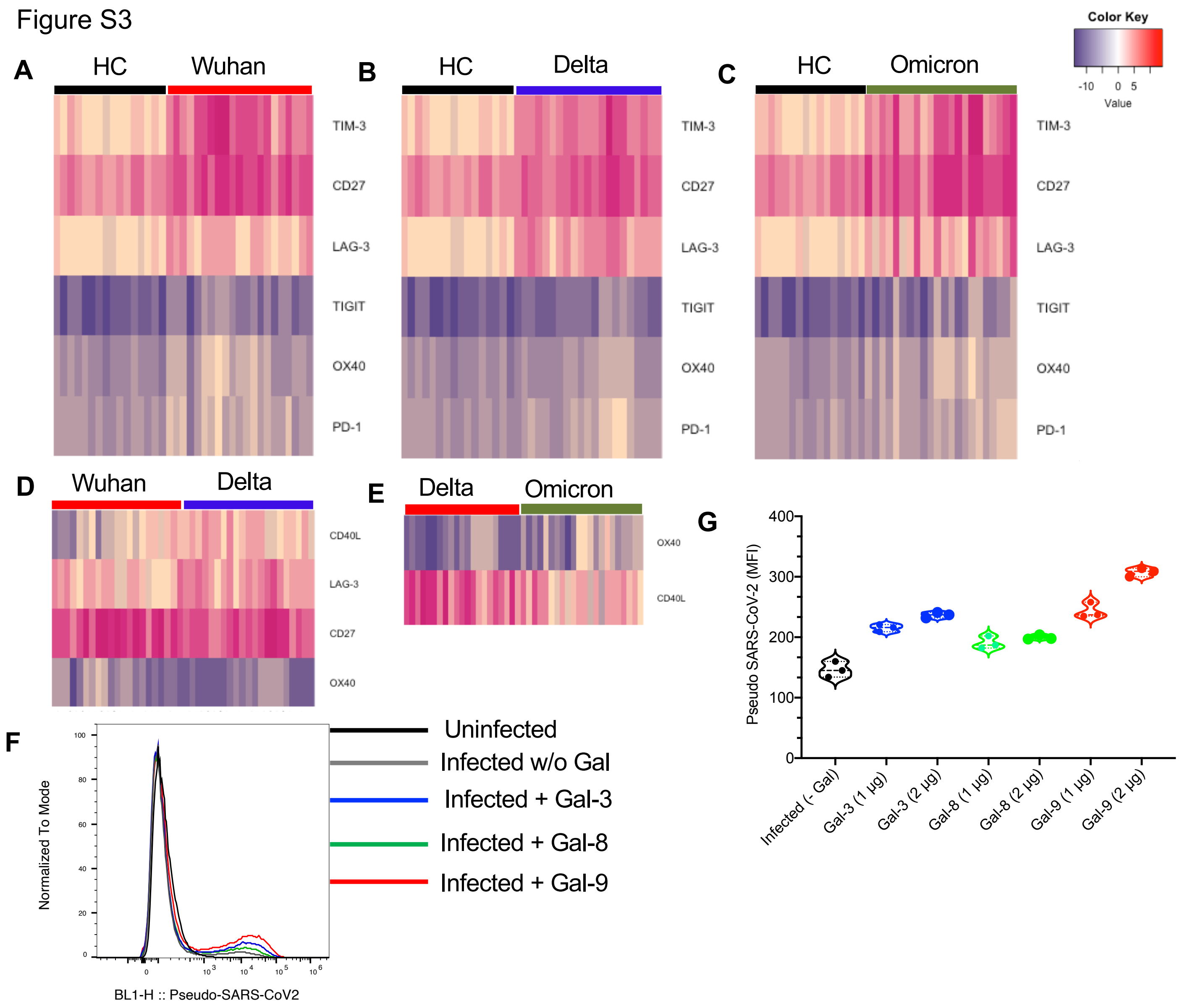

Figure S3. A Heaptmaps showing the concentration of indicated sICs per individual in HCs vs infected individuals with the Wuhan strain. **B** Heaptmaps showing the concentration of indicated sICs per individual in HCs vs infected individuals with the Delta variant. **C** Heaptmaps showing the concentration of indicated sICs per individual in HCs vs infected individuals with the Omicron variant. **D** Heaptmaps showing the concentration of indicated sICs per individual in infected individuals with the Wuhan strain vs the Delta variant. **E** Heaptmaps showing the concentration of indicated sICs per individual in infected individuals with the Delta vs Omicron variant. Each bar represents a sample from an individual.
