# Supplementary material for: Analysis of SARS-CoV-2 isolates, namely the Wuhan strain, Delta variant, and Omicron variant, identifies differential immune profiles
Source: Microbiol Spectr. 2023 Sep 7;11(5):e01256-23. doi: 10.1128/spectrum.01256-23 (PMC10581158; doi:10.1128/spectrum.01256-23)
Supplement: Supplemental Table 1 — S Table 1 [file spectrum.01256-23-s0002.pdf]

Table S1. Demographics and baseline clinical characteristics of COVID-19 patients

Characteristics	Patients #	Patients %	Patients #	Patients %	Patients #	Patients %
Males	28/46	60	29/47	61.7	29/47	61.7
Total deceased	4/46	8.7	3/47	6.4	0/47	0
Male deceased	4	100	3	100	1	2.1
Hypertension	2	4.3	3	6.4	4	8.5
Type 2 diabetics	6	13	5	10.6	6	12.7
Dyslipidemia	5	10.8	4	8.5	3	6.4
Asthma	4	8.7	4	8.5	5	10.6
COPD	0	0	1	2.1	1	2.1
Cardiovascular disease	5	10.8	4	8.5	3	6.4
Cancer	2	4.3	1	2.1	1	2.1
EtOH use disorders	3	6.5	4	5.5	3	6.4
Obesity	6	13	5	10.6	7	14.8
Pneumonia	4	8.7	5	10.6	2	4.25
Anemia	3	6.5	2	4.25	2	4.25
Hypothyroidism	1	2.1	0	0	1	2.1
Respiratory support	46	100	100	47	47	100
Chronic liver disease	1	2.1	2	4.25	1	2.1
Ventilation	22	47.8	24	51	9	19.1
Brain ischemic disorders	1	2.1	0	0	0	0

COPD (Chronic pulmonary obstructive disease). Red (Wuhan) blue (Delta) and green (Omicron) cohorts.
